# Machine Learning Prediction of Suicidal Ideation in Community-Based Older Adults using Deep Phenotypes

**DOI:** 10.1192/j.eurpsy.2025.683

**Published:** 2025-08-26

**Authors:** E. Moon, H. Lim, K. Kim

**Affiliations:** 1 Psychiatry, Pusan National University Hospital, Busan, Korea, Republic Of

## Abstract

**Introduction:**

Suicide is a major public health concern, especially among older adults. Early identification of individuals at risk of suicide is crucial for early intervention, which significantly improves prevention efforts. Early identification of individuals at risk of suicide is crucial for prevention.

**Objectives:**

This study aimed to develop a model for predicting suicidal ideation in community-based older adults using deep phenotype data with machine learning classifiers.

**Methods:**

A study investigating suicidal ideation in community-based older adults utilized a mobile assessment bus to collect data from 358 participants. Deep phenotype data, including Patient Health Questionnaire-9 (PHQ), Generalized Anxiety Disorder-7 (GAD), World Health Organization Quality of Life (WHOQOL), Perceived Stress Scale-10 (PSS) questionnaires, and 32-channel EEG recordings using the 10/20 system, were acquired. Of these participants, 238 completed all assessments. Suicidal ideation was defined by a score of 1 or higher on the ninth question of the PHQ-9. Data from both groups were compared, and features with an effect size of 1 or greater (Cohen’s D) were selected for further analysis. Cohen’s D. Machine-learning classifiers, including Support Vector Machine (SVM), Random Forest (RF), and Linear Discriminant Analysis (LDA) were employed to predict suicidal ideation using a 7:3 training-test split repeated 100 times to obtain performance metrics.

**Results:**

Scores on the PHQ, GAD, and WHOQOL scales differed significantly, while the PSS data showed variations in all items except one between the group with suicidal ideation and the group without. Notably, analysis of the EEG data from eight brain regions identified disparities in 108 out of 248 features. Among all data, ten features with Cohen’s D values exceeding 1 were identified, primarily consisting of questions directly related to themes of negative emotions. Using these features, the classification model achieved an AUC of 0.8913, demonstrating strong predictive performance for suicidal ideation.

**Conclusions:**

Our findings demonstrate the potential of deep phenotyping, even in community-based settings, to predict suicidal ideation in older adults. These insights can inform the development of suicide intervention systems. Additionally, refining predictive models to encompass broader mental health symptoms could solidify deep phenotyping as a crucial tool for early intervention in public healthcare.

**Disclosure of Interest:**

None Declared

